# Rare case-series of electrocautery burn following off-pump coronary artery bypass grafting

**DOI:** 10.5249/jivr.v6i1.456

**Published:** 2014-01

**Authors:** Feridoun Sabzi, Mojtaba Niazi, Alireza Ahmadi

**Affiliations:** ^*a*^Imam Ali Heart Center, Kermanshah University of Medical Sciences, Kermanshah, Iran.; ^*b*^Department of Anesthesiology, Imam Reza Hospital, Kermanshah University of Medical Sciences, Kermanshah, Iran.

**Keywords:** Electrocautery, Complication, Burn

## Abstract

With an increasing number of off-pump coronary artery surgery procedures in high-risk patients with coagulopathy, including renal failure, hepatic failure and anticoagulant drug-using patients, the frequency of related complications such as repeated exploration for bleeding is also increasing. The associated co-morbidity and repeated use of electrocautery in postoperative bleeding leaves patients susceptible to electrocautery ulcers. In this case series, rare cases of cautery burn with unique causative mechanisms are described.

## Introduction

Electrocautery ulcer is a very rare intra-operative accident, with an overall incidence of one patient in 1,000 cardiac surgeries. Despite the rarity of this complication, the causative mechanism is interesting, instructive and unique. This article presents these rare case series of electrocautery burn along with literature reviews for conducting a thorough electrocautery burn study. The electrocautery devices unit is frequently concerned about intra-operative skin sores. In many cases, the injury may be caused by a fault in the electrosurgical unit or its connection or a malfunction of that device.^1,2,3^ The causes of skin burns in the operation room (OR) include: 1- Radio frequency in electro surgery, 2- Thermal injuries as result of direct contact with heating pads, diathermy, electrocautery, flash-sterilized surgical instruments, heated probes, radiant warmers, exam and operating lights, fiberoptic light cables, lasers, Povidone-iodine prep solutions and Ethylene oxide by improper aeration of Ethylene oxide-sterilized devices.^4,5,6^ Among the above-mentioned factors, burns that occurred in the cardiac OR were limited to some special factors, most of which related to staff and surgeon ignorance. Depending on the time of the occurrences and location of the burn, the equipment, devices, and solutions that may have to be inspected include: the electrocautery device and its components including the hand piece electrodes, grounding electrodes, cables, and conductive gel, hypo/hyperthermia units with associated blankets and patient rectal or nasopharyngeal temperature probes, heating pads, heat lamps, radiant warmers; diathermy units; transcutaneous oxygen and carbon dioxide electrodes; pulse oximeter probes; bispectral index electrodes; intra-aortic balloon pumps and their accessories. These accessories include electrodes, cables, tubes, tourniquets, monitors such as electrocardiogram (ECG), electroencephalogram (EEG), and temperature with associated cables, electrodes, and probes, cardiopulmonary bypass equipment; operating room tables; anesthesia masks and tubing; prepping and antiseptic agents; ointments; and linens and allergic reaction to adhesives, electrode gel, ointment, and skin prep solution likes povidone iodine and deconex. But the most important device that predisposes the patient to intraoperative burn is the electrocautery unit.^6,7,8^

**Operation technique**

Saphenous vein grafts and left internal mammary artery (Lima) were always anastomosed to the left anterior descending artery. Intra-operative monitoring of patients undergoing off-pump coronary artery bypass graft(CABG) surgery typically includes a combination of the following: arterial pressure monitoring, electrocardiographic monitoring and sometimes transesophageal echocardiography to detect wall motion abnormality or valve incompetence. Anesthesia management includes use of short-acting anesthetic drugs, pain control, one-lung ventilation to improve access and reduce lung movement by inflation and deflation of the left lung. Sometimes, for this goal, we have a fixed pericardial edge to left sternal border. Conventional immobilization techniques such as deep pericardial sutures were used to provide better access to lateral and posterior target vessels. Sometimes two or four sponges were placed under the heart. Others immobilization techniques include use of intraoperative Esmolol to induce bradycardia and help reduce the technical difficulty of performing surgery on a beating heart. Whenever indicated, the patient position was changed to a slight right lateral decubitus and 20-30 degree Trendelenburg. A bypass circuit was not settled but a perfusionist was available in the OR. Heparin 100 mg/kg was administered to keep the activated clotting time (ACT) between 200-400 seconds. During the operation, an auto transfusion device was not used for blood recovery. The two stabilization techniques involved use of either a suction or compression device: suction stabilization lifts the epicardium and pulls the tissue target to the immobilized target area. Compression stabilizers push downwards to compress the myocardium or restrict its motion. Before anastomosis, the target coronary artery is temporarily occluded proximally and distally by fine bulldog clamps or looped 5/ 0 Vilene suture. If the systolic bland pressure was reduced to 70 mmHg or less an inter-luminal shunt was used. Phenylephrine (1-2 mg) was administered intravenously to the patients whose hemodynamic status was unable to keep the blood pressure between 70-90 mmHg

## Case report 1

**Figure 1:**
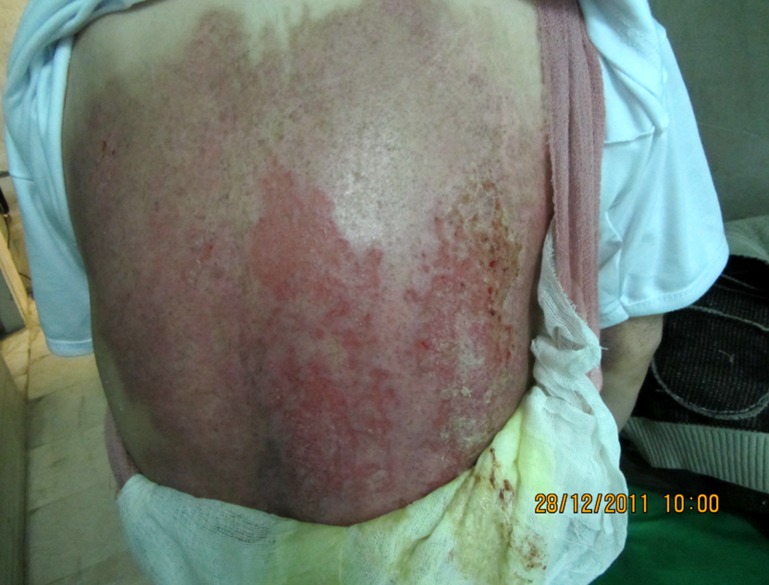
Photograph of the burn site on the day of surgery

**Figure 2:**
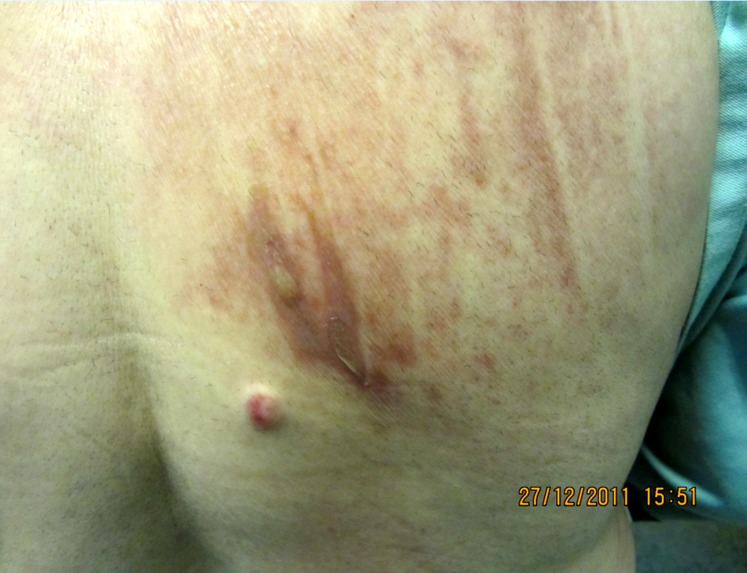
Photograph of the buttock burn site on the day of surgery

The patient was a 56-year-old man with a medical history notable for coronary artery disease, diabetes, and a past medical history of spinal cord fracture repaired by external fixators. Of note, several titanium plates remained in his spinal columns following traumatic spinal cord fracture treated by plate and screw application five years ago. He had off-pump coronary artery bypass grafting. Accordingly, he underwent re-exploration for excessive postoperative bleeding, and he was placed in the supine position with his head resting on a well-padded gelatin horseshoe. One grounding pad was applied on the left arm. His surgery proceeded without incident. Total operating time was 2.5 hours for CABG and one hour for re-exploration for bleeding. When he was turned over at the conclusion of the second surgery for ICU transferring, full skin erythema was noted on all of his back and sacrum. A swollen 3 × 3 cm round area of brown and black dermis with visibly coagulated veins was noted above his T6 and T7 spinal column. (Figure 1). Two zones, the inner black and outer brown, extended concentrically from this area of burns and corresponded to the exact location of his indwelling plate and screw hardware as seen on radiography. The burn was treated with zinc oxide gel. Follow-up at two months revealed complete healing of the burn site (Figure 2).

## Case report 2

A male patient underwent an uncomplicated off-pump CABG that took approximately two hours to complete. A ground-referenced electrosurgical unit was used for the procedure.

An ECG monitor was connected to the patient. The electrocautery unit was set up by the circulating nurse, and the device was initially set at 3 in the cut and coagulated mode. The device setting was increased from 3 to 4 after the surgeon noticed inadequate surgical effect and requested more. Before the request for the increased dial setting, an anesthesiologist tripped on the grounding electrode cable. The grounding electrode was applied to the middle left arm after supine position for the procedure. The electrode was not checked for proper contact with the patient following repositioning. After the procedure, when the drapes were removed from the patient, it was noticed that the conductive portion of the paddle electrode was not completely in contact with the patient. There was no injury beneath the dispersive electrode. However, postoperative skin check in ICU revealed an area of skin injury behind the patient's sacrum, which had been in contact with a wet operating room cover. The injured area was a light cream color, was hard to the touch, and had a red rim. It measured approximately 1.5 x 3 cm. When the operating room staff member tripped on the cable, the dispersive electrode was pulled off the patient. Because the electrical cautery grounding pad for electrosurgical currents was weakly connected to the patient, the electrosurgical currents found an alternate pathway through the patient/wet table cover. The dial setting was increased to overcome the resistance offered by the patient's skin in contact with a small part of table via the wet cover. Current flow through this small area of patient contact caused tissue damage at this site. After the patient has been established in the desired position, every repositioning or request from the surgeon for an increase to the dial setting or ineffective cautery in coagulation mode or any interfering of device with ECG monitoring or Intra aortic balloon pump machine should be accompanied by a warning about electrocautery malfunction. The circulating nurse should not automatically increase the dial setting after a request from the surgeon. Before doing so, the nurse should check all cables and connection of electrical cautery grounding pad. And after checking the cables and devices, the nurse should increase the dial setting as little as possible. 

## Case report 3

A 55-year-old patient with a history of coronary artery disease underwent CABG. The surgery lasted three hours. An electrical cautery grounding pad and a monitoring device with cables and accessories were used throughout the procedure. The cautery dial was initially set at 3 in the coagulated mode and 3 in the cutting mode. The coagulation dial setting was increased from 3 to 5 at the surgeon's request during sternum closure. The dispersive electrode was applied to the patient's left arm and was found intact at the end of the procedure. Because this was emergency surgery, the surgical prepping solution had been poured on the patient, and it had pooled underneath him on the operating table. Eighteen hours after surgery, blistering skin lesions were found on the patient's sacral area and adjoining upper buttock area. This lesion area measured approximately 7 x 10 cm, was red across its entire surface with blisters beginning to form, and felt warm and firm in and around the involved area. Further immobility during recovery also contributed to the development of the lesions, as did the wetness from the pooled prep solutions. 

## Discussion

The use of cautery dates back to ancient times. Egyptian papyrus scrolls reveal the use of heated stone for traumatic injury treatment.^9^ In the West, many credit William Bovie as the father of electrosurgery devices. However some believe that scientific and workable advancement of cautery had been known for hundred years before Bovie was active.^10^ Bovie’s preliminary electrosurgical device causes tissue to heat rapidly to high temperature that in turn causes blood coagulation and control of bleeding at the cost of burning some surrounding tissue. Electrocautery burns are often the result of exposure of the patient's skin to a high-frequency electrical circuit. With modern electrosurgical equipment that is able to selectively cauterize tissues by scattering current density as it leaves the patient's body, this is very rare. Modern cautery is accomplished by a paddle electrode or indifferent or grounding electrode that completes the looped circuit between the patient and electrocautery device. Since the application of the first successful unipolar electrocautery by Bovie in 1928, and the bipolar electrocautery by Gendron in 1939,^11^ Goldwyne et al.^12^ and Zinder et al. have reported a number of thermal complications in skin, bone and other organs.

The electrocautery unit generates two types of electrical current, depending on the desired mode. A cutting current is produced by a vacuum tube oscillator producing a sinusoidal wave form and this is used in skin incision. The most frequently used mode is coagulating current that is produced by a spark-gap circuit resulting in a damped oscillating wave at an average frequency of 1 600 000 cycles per second (Hz), several hundred volts being applied to the spark gap. The average out-put to the hand piece electrode is 400 watts, but may exceed 800 watts if the tip of the hand piece has debris. Debris and coagulated and necrotic tissues cause unusual impedance to active electrode current flow. The spark-gap mechanism generates an electromagnetic wave which can be picked up as a parasite by electronic monitors and mobiles, computers and even radio receivers in proximity to the OR. Potentially deleterious effects on the heart are obviated by the very high frequency of the current. The power output of the electrocautery unit is purposely concentrated in the active electrode. The body skin usually has a high resistance; an average of 4000 ohms to a usual current flow of 400 or 600 watts.^13,14,15,16^

Basically, a monopolar electrosurgical current in a monopolar circuit functions in the following manner: an electrosurgical current is produced in a generator, and this is led to the electrode through cables. This hand piece or active electrode transmits the device energy as an electrical current to the tissue, and the current will leave the patient through an electrical cautery grounding pad and then will return to the generator. Since an appropriate connection of the electrical cautery grounding pad to the skin has a much greater surface area than the small tip of the active electrode, the current is dispersed over a large area, thereby minimizing the heating of the tissue underneath the electrical cautery grounding pad. However, Fickling et al.^17^ have shown that if the surgeon uses the active electrode or hand piece for a prolonged time period, with increasing current, the temperature of the paddle that is in connection with the patient will also increase because the electrical cautery grounding pad cannot disperse the current on a larger surface. According to the experimental work of Fickling et al, if the temperature of the electrical cautery grounding pad, as the result of prolonged cautery use, exceeds 44ºC, burns may occur.^17^ Makama et al.^18^ stated that multiple factors contribute towards such burns. Firstly, an interruption or prolonged activation of active electrode or hand piece, especially at high voltages, would heat up the electrical cautery grounding pad excessively. Secondly, if the contact area under the grounding pad were to decrease, this would result in concentrating the current only at the remaining contact points and thus the current would not disperse across the whole area of the grounding pad. Improper dispersing of current could occur for the following reasons. The grounding pad may have been placed on: an area with little muscle such as a bonny prominence (elbow joint, lower forearm,); areas with a lot of body hair (hairy forearm, unshaved thigh); areas with little soft tissue (lower legs). The use of oily cream instead of conductive cream, on areas with previous scars^19,20,21,22,23,24^ can also lead to improper dispersing. We believe that the most important factor predicting postoperative burn is the use of high currents for long periods of time without interruption. This is done in order to reduce postoperative bleeding, during exploration for postoperative excessive bleeding in a valvular or CABG operation. The other complications of electrocautery are interference with the monitoring of ECG and pulse oximeter and bispectral index, malfunction of pacemaker, implanted implantable cardioverter defibrillator (ICD, burn injury, intra organ fire, and inhalation of diathermy smoke and gene mutation. Burn injuries in the operation room occur from cautery, warming devices and airway fires.^22,23,25,26,27^

Cautery fires constitute up to 20% of burn claims. This study aims to evaluate electrocautery burn and compare it with the pressure sore that occurs after coronary artery bypass grafting with off-pump method. Intraoperative electrocautery burns can occur following these technical and equipment faults: direct osculation; high voltage use; continuous or interrupted operator use of the electrocautery; high resistance of active electrode tip by coagulated or necrotic tissue; improper attachment or placement of paddle; burns resulting from electrode heating of pooled solutions such as patients sweating, iodine solutions, intraoperative irrigation; iatrogenic injection of solution in the joint in orthopedic surgery and burns occurring as a result of circuits generated between the active electrocautery tip and a subsidiary grounding source.^24,25,28,29,30,31,32,33^

A subsidiary intraoperative circuit utilized previously placed external fixator in the patient's spinal column as the grounding electrode, resulting in a small full-thickness skin necrosis overlying the site of plate implantation and heating of the pooled solution caused second degree burns to the patient’s back. We recommend the reduction of this type of electrocautery burn by avoiding paddle placement on the hairy forearm, on bonny prominence, and on a place with little soft tissue, although further investigation is needed to determine optimal grounding electrode placement with respect to known indwelling hardware such hardware in legs, arms spinal cord, thigh, skull and pelvis. In contrast to electrocautery burn, over one hundred risk factors for pressure ulcer development are reported in the literature. Kemp et al,^34^ measured seven variables and the risk factors from the Braden scale. They showed in their study that age, time on operating room and cardiopulmonary bypass (CPB) usage are significant risk factors for pressure ulcer development. The Braden scale was not a significant predictor of pressure ulcer development. In Papantonio et al’s^35^ prospective study, 136 adult cardiac surgery patients were evaluated for development of pressure ulcer. 

Intra aortic balloon pumps (IABP) were used in all of them and all patients were lying on low-air loss beds. In this study,^13^ variables were examined as possible risk factors for Pressure Ulcer (PU) development. Factors predicting development of PU were included chronic obstructive pulmonary disease (COPD), aging, diabetes reduced hematocrit, hypoalbuminemia ecchymosis and prolonged operating time. 

Jesurum et al’s^36^ study included patients underwent open-heart surgery with IABP lying on low-air-bed. Variables were examined as risk factor for the development of PU. Factors predicting development of PU on first post-operative day were: age, stroke, renal failure and high APACHE. Results of this study revealed that pressure sore is a patient-dependent complication and in contrast to electrocautery sore which is related to technical or device faults and to experience and care of OR staff.

## Conclusion

Complete evaluation of a skin injury includes consideration of all possible device faults or body-preparation solution interactions. It must also consider the possibility that the injury may be a pressure sore, electrocautery ulcer, thermal injury or reaction to drugs or antiseptic solution. Some investigators assumed that an electrocautery unit or solution caused the injury simply because it was used. It seems that most of these assumptions are often incorrect and preclude considerations of other possibilities, and may bias the studies. When evaluating a sore in operating room, the medical engineer and surgical staff team must make sure that all possibilities and probabilities are considered and that surgeon and staff involved in the incident are questioned. Only then will it be possible to develop effective preventive measures.
